# Sociality and Wild Animal Welfare: Future Directions

**DOI:** 10.3389/fvets.2019.00062

**Published:** 2019-03-19

**Authors:** Philippa Brakes

**Affiliations:** Adaptive Rationality Group, Centre of Ecology and Conservation, University of Exeter, Penryn, United Kingdom

**Keywords:** sociality, animal welfare, social learning, animal culture, social structure, vertebrates, five domains model, wildlife

## Abstract

Emergent evidence of aspects of sociality, such as social structure and social learning, across many vertebrate taxa, warrant more detailed consideration of their influence on welfare outcomes for wildlife. Sociality can be dynamic across organismal development, it can: provide protection through safety in numbers; may influence breeding outcomes via mate choice and alloparental care; can influence foraging success through transmission of social information and co-operation; and it can provide opportunities for the spread of novel behavior. Social learning itself provides an important mechanism for resilience in changing environments, but also has the potential to increase vulnerability or facilitate the spread of maladaptive behaviors. The welfare consequences of vertebrates living in social groups are explored using Wilson's 10 qualities of sociality as a framework, and the implications of human activities are discussed. Focus to date has been on the importance of social networks for the welfare of farmed or captive animals. Here I consider the importance of social networks and sociality more generally for the welfare of wildlife and explore Mellor's five domain model for animal welfare within the context of wildlife sociality.

## Introduction

Sociality is a measure of the degree to which animals interact or form long-term or transient associations and is prevalent across a broad range of vertebrate and invertebrate taxa. Animal welfare, on the other hand, is a multidimensional field, measured through a range of criteria from health and comfort, to the ability to express natural behaviors (1). While some natural behaviors may be considered, many aspects of sociality are often overlooked in animal welfare assessment and this is particularly the case for wildlife, where social settings may be complex.

Wild animal conservation and individual and group welfare are deeply intertwined (2). While the social environment is undoubtedly of significance to both the conservation and welfare of many species, quantifying the welfare implications of aspects of sociality can be challenging in the wild. The social environment has been described as being comprised of non-random and heterogeneous social interactions (3, 4). The social environment of vertebrate taxa is highly diverse, ranging from: the complex third order alliances of male bottlenose dolphins (*Tursiops* sp.) (5); to matriarchal African elephant societies (*Loxodonta* sp.) (6); to the hierarchical group dynamics of flocking birds (7). For long-lived, wide ranging species, such as blue whales (*Balaenoptera musculus*), there are also important spatio-temporal considerations for interpretation of behavior and associations (8).

Aspects of sociality, such as social structure and social transmission of information, across many vertebrate taxa, warrant a more detailed exploration of their influence on individual and group welfare outcomes for wildlife. Quantifying the welfare of wild animals presents a number of unique challenges. Wildlife welfare is often framed in terms of physiology or individual behavior. Nevertheless, better understanding of the processes of social behavior may provide important insights for the welfare of wildlife. While the focus of this review is vertebrates, there are some notable invertebrate taxa, such as cephalopods (9), which might also benefit from similar consideration.

Wilson's 10 qualities of sociality (10) have previously been suggested as a framework for evaluating the importance of sociality for individual and group welfare for cetaceans (whales and dolphins) (11). Here I attempt to expand these ideas to examine how sociality may be important for the welfare of a range of vertebrate taxa: exploring the risks and benefits of sociality to animal welfare; investigating the relationship between sociality and the impacts of human-induced rapid environmental change (HIREC) (12) on sociality and animal welfare. I then examine this new perspective within the context of the five domains model (13) of animal welfare.

## Sociality and Living in Groups

There are a number of potential ecological and animal welfare benefits from living in groups which include: predator defense, cooperative foraging, mating opportunities, and reduced vulnerability to infanticide (14). However, there are also risks and costs associated with group living, such as: increased risk of spread of disease, or increased conspicuousness to predators and competition for resources (3). Living in groups can also influence individual reproductive fitness, gene flow and spatial distribution (10, 15) and it has been argued that the buffering effects of social support may be relevant to farm animal welfare (16). There are likely many facets of sociality which have implications for both individual and group welfare in wildlife.

E.O Wilson listed 10 “qualities” of sociality (10) which are used to understand and classify conspecific groups. These characteristics of animal societies have also been used to provide a framework for evaluating the importance of sociality for individual and group welfare in cetaceans (whales and dolphins) (11). Here this framework is used to examine Wilson's 10 qualities of sociality within the context of wildlife welfare, more generally (Table 1).

**Table 1:** Wilson's 10 “qualities” of sociality (10) and welfare considerations for wild animals [adapted from Brakes (11)].

**Quality**	**Welfare considerations**
Group size	Welfare benefits may include predator defense, co-operative foraging, mating opportunities, and reduced vulnerability to infanticide (14). Here “group” is defined as “animals that actively achieve or maintain spatiotemporal proximity” (after 13). However, some of these welfare benefits may also be obtained in aggregations, which are not the result of social interaction but instead result from patchy resource distribution.
Demographic distribution	Populations and social groups may to some extent be robust to fluctuations in demographic distribution (from a welfare perspective), but this may depend on the extent and duration of parental and alloparental care and the social role of older individuals in predator defense, resource acquisition or as repositories of social knowledge (6, 17).
Cohesiveness	Wilson (10) suggested that the proximity of individuals may be used as an index of sociality. Today the more common measure is the rate of interactions (18). If the rate of interactions correlate with social behaviors, such as cooperative foraging, then it may follow that successful feeding could be correlated with interaction rate.
Patterns of connectedness through communication	Communication is central to sociality. Many vertebrate species exhibit complex patterns of connectedness through communication; these can be vocal, visual, tactile, or chemical. Some aspects of communication, such as bird song, can be socially transmitted, and the transmission itself may be dependent on aspects of the social network of the population, which may also extend between species (19). Measuring patterns of connectedness through communication may not always be straightforward, e.g., there is evidence that dolphins may eavesdrop on the echolocation of others (20). In cases where communication involves the transmission of social information, this may be relevant to wildlife welfare, for example where it relates to resource acquisition, such as food patches.
Permeability of movement between social groups	The movement of individuals between groups may act as vectors for the spread of disease or information. In addition, permeability has implications for the transmission of information between social groups, e.g., information on predators or resource acquisition.
Distinct social units	Potentially relevant to the emergence of unique socially learnt behaviors and cultures. Resilience to environmental change may depend on a variety of aspects of sociality, such as group composition (6) and how likely individuals are to innovate, or for innovations to be transmitted in the face of external pressures (21, 22).
Differentiation into social roles	Female pilot and killer whales exhibit a post-reproductive phase, indicating an import role within their social groups (17). This is supported by evidence that post-reproductive female killer whales boost the fitness of kin (23). The removal of individuals with key social roles, such as matriarchs, may have welfare repercussions for their social group (24, 25).
Integration of behavior	Whitehead (18) argues that measuring synchrony may be one way to examine integration of behavior. The welfare implications of synchronous behavior have not yet been extensively examined, but synchrony likely influences energy expenditure while traveling and hunting. It may also be useful to explore how fluctuating asymmetry (FA) (26, 27) varies in relation to synchronous and other integrated behaviors.
Information flow	May be relevant to resilience, particularly in relation to innovative foraging techniques, resources patches, and safe habitat (28, 29)
Time devoted to social behavior	The welfare implications of the proportion of time devoted to social behavior depends on the cost and benefits to the individual of spending time exhibiting that behavior, which may be contingent on the other qualities of sociality identified by Wilson (10).

## Risks and Benefits of Sociality

Within the vertebrate taxa there are a variety of social structures, types of association between individuals, and transmission pathways for social information. As a result the relationship between sociality and welfare across vertebrate taxa is multifaceted (see Table 2). Undoubtedly, sociality provides benefit to individual welfare, for example in predator defense, alloparental care, or by increasing foraging success through the transmission of information about food sources or other types of public information, or through co-operative foraging. But sociality also has associated risks, such as the increased probability of the spread of disease, or conspicuousness to predators. These risks and benefits can change according to the environment and may also be dynamic across the developmental stages of an organism.

**Table 2:** Summary of the importance of social living for wild animal welfare [after Brakes (11)].

**Positive**	**Negative**
Resilience to environmental change and adaptation through social learning and the spread of novel behavior	Conservative cultures may hinder adaptation and resilience Potential for spread of maladaptive, or undesirable behavior through social learning
Individuals may act as repositories of social knowledge for the social group	Vulnerability from removal of repositories of social knowledge or individuals with specific social role
Potential for alloparental care in some species	Vulnerability of some cohorts and dependents if “carers” are removed Potential for increased competition for mates
Foraging cooperation Sharing information on food sources, either directly or through local enhancement	Foraging competition
Predator defense and alerting conspecifics to danger	Conspicuousness to predators
	Generally high probability of disease transmission in higher density groups

While the risk of spread of disease and parasitic burdens associated with living in social groups has been extensively studied, sociality has, nevertheless, evolved independently in many taxa, demonstrating that these risks are overall offset by the benefits of sociality.

### Social Support Hypotheses

The potential benefits of group living are varied (Table 1), but social support may specifically provide beneficial effects to the recipient, irrespective of whether or not the recipient is being challenged; or social partners may modulate or downregulate the impact of stressors on the recipient's homeostasis [a process known as stress buffering (30)]. Stress buffering has been investigated for farm animal welfare and it has been suggested that farmers could exploit animals' natural ability to benefit from their social group to obtain better welfare outcomes (16). Arguably, for some highly social species of wildlife it may also be the case that better management can be achieved through strategic use of elements of animal sociality to enhance welfare outcomes.

### Social Learning, Animal Culture, and Welfare

Social learning—“learning that is influenced by observation of, or interactions with, another animal (typically a conspecific) or its products” (31)—has been observed across a broad range of vertebrate taxa (32) and can be important for conservation efforts (21, 33, 34). Social learning can act more rapidly than the intergenerational process of Darwinian selection (21) and some facets of sociality may also hold important insights for wild animal welfare. For example, social learning can provide mechanisms for resilience to ecological or anthropogenic stressors, such as reduced prey abundance [for example through diversification of foraging strategies (22, 35, 36)]. In contrast, it can also result in the transmission of socially learnt conservative foraging specializations, such as those found in killer whales (*Orcinus orca*), resulting in the species being less, rather than more, resilient to fluctuations in prey abundance (21).

Social learning also has the potential to facilitate the spread of maladaptive behaviors, which can evolve much more rapidly than genetic selection can counter them (21, 37). This is particularly relevant to animal welfare in human-wildlife conflict situations, both in relation to the spread of behaviors that lead to human-wildlife conflict (38) and in relation to facilitating resolution of these conflicts (39, 40). In all cases, a better understanding of the processes of social learning may help mitigation efforts and improve welfare outcomes for wildlife.

Social learning can also result in more persistent behavioral traits developing into “animal cultures.” Whitehead and Rendell (41) define culture as: “information or behaviors—shared within a community—which is acquired from conspecifics through some form of social learning.” But the interplay between animal culture and individual or group welfare within wild vertebrates may be complex. Cultural behavior may shape both social relationships and social structure (42), or even act as a marker of group identity (41, 43). Although social network analyses have been used to examine the welfare implications of disrupting these social systems (44) (see section Tools for Assessing Sociality and Wildlife Welfare), most studies have focused on farm or captive animals and the implications of these aspects of sociality for wild animal welfare warrant further exploration.

## Human Impacts on Sociality and Wildlife Welfare

The impact of human activities on wildlife and habitat is ubiquitous across virtually all ecosystems and HIREC is now a widely acknowledged phenomenon (12). From deforestation, to climate change and ocean acidification, wildlife around the globe is challenged by rapid environmental change, but aspects of sociality may provide opportunities for resilience to this rapid change, or increase vulnerabilities (45). It is timely to examine how sociality may influence wild animal welfare and responses to HIREC.

HIREC has the potential to influence all behavioral domains associated with social behavior, from breeding to communication, foraging and migration. A method to classify this vast array of threats from an individual welfare perspective is to consider acute and chronic implications for sociality and welfare (Table 3).

**Table 3:** Acute and chronic implications of HIREC for sociality and welfare.

**Threat**	**Acute implications**	**Chronic implications**
Deforestation	Disturbance	Displacement
Hunting	Separation of social groups during hunting, potentially resulting in fragmentation	May result in the removal of individuals that have a specific social role (24)
Bycatch and entanglement	Acute suffering associated with being caught in fishing gear	Long-term suffering of individuals who remain entangled in fishing gear for days, weeks, or potentially months, with unknown implications for social interactions
Pollution	Potential to interfere with or mask the transmission of social information (e.g., noise) and/or result in the loss of key individuals	Anthropogenic pollution (e.g., noise, light, chemical pollution etc.) may influence the welfare of entire social groups in a chronic manner, potentially leading to displacement, and/or fragmentation
Harassment or displacement	Loss of cohesion within the social group in short-term	Longer-term loss of cohesion within the social group, particularly if harassment or displacement is persistent
Anthro-dependance^a^	Dependence on a food source provided by humans e.g., crop raiding, which may result in culling	May lead to loss of knowledge of non-anthro-dependant foraging strategies within social groups
Climate change	Potentially multiple implications including disturbance and displacement	Potentially multiple implications including disturbance and displacement
Ocean acidification	Unknown	Unknown

a *Dependence on human activities*.

## Cognition, Sociality, and Wildlife Welfare

Understanding and predicting how individuals behave in response to HIREC may be important for evaluating aspects of sociality relevant to wildlife welfare. For example, understanding how individual innovations arise may be relevant for predicting—and setting up optimal conditions for—the spread of novel behavior through social learning. Wildlife behavioral responses are governed by cognitive processes ranging from perceptual processes to learned behavior. Understanding these cognitive processes and the effect of HIREC associated stress, may also assist in reducing human impacts on wildlife (46). It has been argued, using the same rationale, that if cognition underlies a behavior that is relevant to welfare, then understanding these cognitive processes may help in achieving better welfare outcomes for species such as cetaceans (11) and this is arguably also the case for other wildlife.

Greggor et al. (46) argue that “*cognitive theory can thus help predict how best to manipulate and exploit attentional biases, innate responses, and learning tendencies to enhance conservation efforts*.” For example, they argue that mitigation methods will only be effective if they are reliably perceived by the target species (e.g., preventing birds colliding with human-made structures) and that this perception is rapidly learned. The same argument can been made for a range of wildlife mitigation activities, including: acoustic “pingers” on fishing nets designed to prevent dolphins from becoming entangled and boundaries designed to prevent crop raiding. The success of some mitigation methods may also be contingent on the social transmission of information, which in turn is contingent on other aspects of sociality such as social structure.

## Tools for Assessing Sociality and Wildlife Welfare

Asher et al. (47) argued for expanding the scope and development of new quantitative methods for the analysis of various aspects of behavioral organization as indicators for animal welfare, including: fractal analysis, temporal methods, social network analysis (SNA), and agent-based modeling and simulation. Social network analysis has now begun to emerge as a tool for assessing animal welfare. Kleinhappel et al. (44) argue that SNA is as yet, underused in the field of animal welfare and suggest a number of opportunities for using these methods, particularly in relation to the welfare of captive animals. However, SNA also has important application for assessing the welfare of wild populations.

SNA can be used to detect and describe the patterns and quality of interactions among individuals, with implications for physical health (e.g., disease transmission), psychological health (e.g., stress and social buffering), and social well-being (e.g., group stability) (48). These types of analyses have been used to investigate animal welfare in captive settings [e.g., for captive elephants (49) and primates (47)] and also have value in wild settings. For example, Godfrey et al. (50), demonstrated the link between parasite infection patterns and the connectivity of individual lizards within a population. It has further been postulated that SNA could be used to track behavioral changes and predict and prevent disease outbreak within groups (44).

## Extending the Five Domains Model to Incorporate Sociality in Wildlife

There are several models that might be applicable for expanding our consideration of wild animal welfare in relation to sociality. Hinde's framework for classifying aspects of sociality (51) might for example provide a basis for attempting to maintain optimal conditions of sociality from a welfare perspective within wild populations. However, trying to ensure optimality in the wild is both challenging to define and difficult in practice.

A more realistic approach may be to use the five domains model of animal welfare (13). While generally applied to domestic and captive animals, where some degree of compromise in welfare circumstances is implicit, the five domains model may be a more practical framework for managing welfare issues arising from human impacts on wild animal sociality. It is suggested here that the five domains model may be useful to avoid directly aversive situations resulting from poor management of the sociality of wildlife, for example through the removal of repositories of social knowledge (25). Instead, it may be better to try to manage wild environments so that socially complex systems can thrive and avoid situations that will hinder their development. The five domains model itself resulted from recognition of the need to provide positive experiences for animals (beyond the five freedoms framework) (52) and this approach may be useful for exploring how we can monitor and potentially assist social systems to flourish in the wild.

The objective of the five domains model is to “draw attention to areas that are relevant to both animal welfare assessment and management” and focus on the “presence or absence of various internal physical/functional states and external circumstances that give rise to welfare-relevant negative and/or positive mental experiences, i.e., affects” (13). In formulating the five domains model, Mellor “carefully and cautiously” considered the affects of the first four domains on nutrition, environment, health and behavior, on the fifth domain of mental state.

Considering some of the issues raised in this review, here I have attempted to explore some of the possible implications of sociality for wildlife welfare within the context of the five domains model (Table 4). This list is not intended to be exhaustive, but instead is a first exploration.

**Table 4:** Potential interface between sociality and wild animal welfare as it relates to the five domains model (13) of animal welfare.

**Nutrition**	**Environment**	**Health**	**Behavior**	**Mental state**
Social information on food patches	Predator defense	Higher risk of spread of disease	Local enhancement via conspecifics (for example on critical breeding or feeding habitat, or sources)	Behavioral resonance^a^, potentially resulting in positive emotions and closer affiliations
Safety in numbers during foraging	Thermal advantage associated with group living	Increased risk of transmission of parasites	Social learning of: communication; individual foraging strategies; or co-operative foraging behavior	Spread of emotional contagion, both positive and negative (e.g., fear in response to predators)
Social learning of novel foraging strategies	Response to HIREC: resilience and vulnerability to acute and chronic threats (see Table 3)	Better opportunities for mate choice	Role of individuals within their social group e.g., repositories of social knowledge	Social behavior providing opportunities for play and learning (see section Play and the Five Domains Model)
Co-operative foraging behavior		Potential for alloparental care of offspring	Potential for social learning of maladaptive behavior	

a *Strong and involuntary propensity to automatically synchronize with and imitate behavior of others (53)*.

### Play and the Five Domains Model

The stated objective of the five domains model is to provide opportunities for positive welfare (13) and it follows that one such opportunity might be granted through play in wildlife. Play is variable across species, has a variety of definitions (54, 55) and its proximate mechanisms and ultimate functions are not well understood. Play can indicate good welfare conditions, but can also be an indication of stressful situations, for example resulting in reduced parental care (56). Play has a variety of roles and it has been argued that play is usually the first behavior to disappear when welfare is compromised: when animals are stressed, anxious, hungry or unwell (57, 58). It has been suggested that play may be a reliable indicator of psychological and physical well-being (56). However, using play as a definitive indicator for optimal or good welfare can be confounded by several factors including: the heterogeneity of play; the fact that play can be a coping strategy; factors with no obvious relation to animal welfare can influence intrinsic playfulness within individuals; and the fact there are circumstances in which negative welfare can actually result in more play. Thus, play cannot definitely be considered an indicator of optimal or good welfare (59). But play can also be dynamic across life stages and arguably may be important for positive mental state in some species (see Table 4). For example, it has been argued that play may be socially contagious and therefore capable of spreading good welfare in groups (56).

Play behavior may also be of value in relation to innovation. In addition to having a potentially positive influence on mental states, there is potentially reciprocity between the social aspects of play, innovation and how innovation may then spread in wild populations. Bateson (57) argues that numerous functions for play have been proposed but they are not mutually exclusive and there are indications that those individuals who play most are most likely to survive and reproduce.

Potentially, the reciprocity between play and vital rates may manifest through the social transmission of innovative behaviors. Although fitness outcomes are not always synonymous with welfare outcomes, associations and bonds forged through play may influence the spread of information across a network and the trial and error aspects of play may help individuals accumulate latent information, which may facilitate innovation as their environment changes. Thus, it can be argued that play may be an important part of the behavioral repertoire of some wildlife for maintaining resilience.

## Conclusion

The interplay between sociality and the welfare of wildlife is multifaceted. The widespread evolution of sociality, social learning, and in some instances even animal culture, among vertebrate taxa is evidence that overall the benefits of social living out-weight the costs. Nevertheless, the individual and group welfare issues associated with the processes of social living are undoubtedly important for the consideration of wild animal welfare.

Previous explorations of the interface between sociality and animal welfare focus predominantly on domestic or captive animals: an entirely different paradigm from evaluating the implications of sociality on free ranging vertebrate organisms. There are many avenues for further research in this field (16), including for example, capitalizing on the mechanisms of social learning for the restoration and rehabilitation of wildlife populations (53, 60). But perhaps the most pressing are instances where human activities significantly disrupt social systems and can influence individual and group welfare (24, 25).

The principle objective of the five domains model is to move beyond an animal welfare focus of survival, toward individuals thriving. In order to meet the objective of thriving wild vertebrate populations, it is essential to incorporate aspects of sociality into their welfare assessments.

## Author Contributions

The author confirms being the sole contributor of this work and has approved it for publication.

### Conflict of Interest Statement

The author declares that the research was conducted in the absence of any commercial or financial relationships that could be construed as a potential conflict of interest.
